# The Foundling Hospital in Florence

**Published:** 1910-04-23

**Authors:** 


### THE FOUNDLING HOSPITAL IN FLORENCE.

BY A SPECIAL CORRESPONDENT.

The Ospedale dell' Innocenti was the first Foundling Hospital to be established in Europe. Leonardo Aretina was the earliest benefactor, but shortly after its foundation in 1421 it was put under the protection of the "Guild of Silkworkers." These Florentine Guilds corresponded more or less to our Livery Companies, and many of them were exceedingly wealthy and powerful. The outside of the hospital in the Piazza Santa Annunziata is well known. It is a large building with fine arcades, and in the spaces between each arch are the famous Della Robbia medallions, fascinating swaddled Bambini, modelled in blue and white terra-cotta. The building itself was designed by Brunelleschi and his pupil, Francesco della Luna, and is a fine piece of domestic architecture of the best Florentine period.

On entering the courtyard we were most courteously received by the director, who explained all the details of the organisation and arranged for us to visit the hospital. We began with the receiving room, where the infants are first brought, weighed, washed, dressed, and allotted a medal. This medal has a design of our Blessed Lady with Cherubs upon it, a gilt one for the legitimate children, a nickel one with the same design for the illegitimate infants.

Though the institution was founded in the first place for illegitimate abandoned children, yet now under certain conditions the children of poor respectable parents are admitted, maintained for a year, and then returned to their parents. Abandoned children are frequently left at the door, and it is often impossible to trace their parentage, and this institution has been sometimes condemned on account of the facility with which this may be done, some people holding that it is actually immoral to provide mothers with an inducement to abandon their illegitimate infants; but unfortunately the absence of such provision does not prevent infantile desertion. Moreover, at this institution search is always made for the father, and if he can be traced he is compelled to pay for their maintenance.

About 1,100 children are admitted every year, and the average mortality is about 148, a proportion accounted for by the fact that many of them are found moribund, both from their exposure to want and cold. Attached to the hospital is a most complete vaccination department. It is divided into two parts, each part being quite separate. The stalls for the calves are at one end of the hospital garden, and the vaccine-preparing room, bacteriological room, and the hall for public vaccinations are on the ground floor of the hospital.

There is a book kept in which the number of every vaccinated calf is entered, with the morning and evening temperature of the calf on the days that precede and follow the inoculation, and every imaginable detail necessary to ensure perfection is entered too, such as the bacteriological researches made during the different periods of the glyceric mixture, and of the result of the human vaccination with this same mixture. The vaccine here is as perfect as human ability and perfect asepsis can make it, and it is sent in large quantities at a very low cost to other hospitals, institutions, regiments, and even to foreign countries.

In another room, on the ground-floor, milk, ready sterilised and prepared for infants at different ages, is for sale in sealed bottles, and is supplied at a very low cost to any poor mother who likes to come for it.

We next went round the wards, which are quite charming and admirably managed. They are very up-to-date, built with rounded corners so that no dust can collect.

The premature babies are reared in incubators, and every effort is made to fan the little spark of life. Most of the infants are breast-fed, their foster-mothers being obtained from the Maternity Hospital close by. They are well paid and admirably looked after in matters of hygiene. We were shown large baths in which they are required to bathe, and they are provided by the hospital with clean cotton overalls, which they are always obliged to wear. They take the air in a large garden attached to the hospital, and in fine weather take the babies there for an airing too. They are seldom allowed to go into the town, and then only under strict surveillance. They stay mostly about a year, and generally suckle two babies, who are frequently weighed, and their foster-mother changed if they do not thrive.

It is a pleasant sight to see eight or nine of these women sitting in a row feeding their babies, and discussing their "points," each woman firmly convinced that *her* baby is vastly superior to the others.

When the infants are old enough, *i.e.* at two or three years old, they are boarded out with respectable families in the country, and this arrangement gives excellent results, and is surely a far more normal and reasonable life than the routine of a large "orphan school" or "infant asylum." The children become almost one of the family, and are generally deeply attached to their foster-parents. On arriving at years of discretion the boys are generally drafted off into the Army or Navy, if physically fit; the girls are either apprenticed to some trade, or go into domestic service, or if they become betrothed with the authorities' consent, both the trousseau and a substantial "dot" are provided by the hospital. So that from the moment of a child's arrival until his entry into the great outside world, no pains or trouble is spared to make these children (often of poor stock physically) into useful and self-respecting citizens.

